# Molecular Relationships among Obesity, Inflammation and Intervertebral Disc Degeneration: Are Adipokines the Common Link?

**DOI:** 10.3390/ijms20082030

**Published:** 2019-04-25

**Authors:** Clara Ruiz-Fernández, Vera Francisco, Jesus Pino, Antonio Mera, Miguel Angel González-Gay, Rodolfo Gómez, Francisca Lago, Oreste Gualillo

**Affiliations:** 1 SERGAS (Servizo Galego de Saude) and IDIS (Instituto de Investigación Sanitaria de Santiago), The NEIRID Group (Neuroendocrine Interactions in Rheumatology and Inflammatory Diseases), Santiago University Clinical Hospital, Building C, Travesía da Choupana S/N, 15706 Santiago de Compostela, Spain; clararf94@gmail.com (C.R.-F.); jesus.pino.minguez@sergas.es (J.P.); 2 SERGAS (Servizo Galego de Saude), Santiago University Clinical Hospital, Division of Rheumatology, Travesía da Choupana S/N, 15706 Santiago de Compostela, Spain; antonio.mera.varela@sergas.es; 3 Epidemiology, Genetics and Atherosclerosis Research Group on Systemic Inflammatory Diseases, Universidad de Cantabria and IDIVAL, Hospital Universitario Marqués de Valdecilla, Av. Valdecilla, 39008 Santander, Spain; miguelaggay@hotmail.com; 4 Musculoskeletal Pathology Group. SERGAS (Servizo Galego de Saude) and IDIS (Instituto de Investigación Sanitaria de Santiago), Research Laboratory 9, Santiago University Clinical Hospital, 15706 Santiago de Compostela, Spain; rodolfobahamonde@gmail.com; 5 Molecular and Cellular Cardiology Group, SERGAS (Servizo Galego de Saude) and IDIS (Instituto de Investigación Sanitaria de Santiago), Research Laboratory 7, Santiago University Clinical Hospital, 15706 Santiago de Compostela, Spain; francisca.lago.paz@sergas.es

**Keywords:** adipokines, adiponectin, adipose tissue, annulus fibrosus, immune system, intervertebral disc degeneration, leptin, metabolism, nucleus pulposus, obesity

## Abstract

Intervertebral disc degeneration (IVDD) is a chronic, expensive, and high-incidence musculoskeletal disorder largely responsible for back/neck and radicular-related pain. It is characterized by progressive degenerative damage of intervertebral tissues along with metabolic alterations of all other vertebral tissues. Despite the high socio-economic impact of IVDD, little is known about its etiology and pathogenesis, and currently, no cure or specific treatments are available. Recent evidence indicates that besides abnormal and excessive mechanical loading, inflammation may be a crucial player in IVDD. Furthermore, obese adipose tissue is characterized by a persistent and low-grade production of systemic pro-inflammatory factors. In this context, chronic low-grade inflammation associated with obesity has been hypothesized as an important contributor to IVDD through different, but still unknown, mechanisms. Adipokines, such as leptin, produced prevalently by white adipose tissues, but also by other cells of mesenchymal origin, particularly cartilage and bone, are cytokine-like hormones involved in important physiologic and pathophysiological processes. Although initially restricted to metabolic functions, adipokines are now viewed as key players of the innate and adaptative immune system and active modulators of the acute and chronic inflammatory response. The goal of this review is to summarize the most recent findings regarding the interrelationships among inflammation, obesity and the pathogenic mechanisms involved in the IVDD, with particular emphasis on the contribution of adipokines and their potential as future therapeutic targets.

## 1. Introduction

Intervertebral disc degeneration (IVDD) is a chronic, complex and multi-factorial musculoskeletal disorder characterized by metabolic and structural changes that progressively lead to the loss of mechanical stability and shock absorber function of the intervertebral disc [1]. Hence, IVDD is an important cause of low back pain [2]. It is estimated that around 20% of teens have mild-degenerated discs and that 80% of the population suffers from back pain at some point in their lives, being the most limiting factor of activity for people under 45, with a younger trend [3]. Back pain is also a frequent cause for visits to the hospital, absence from work, hospitalizations, and surgical procedures [3]. Regardless of the high IVDD socio-economic impact, its etiology, progression and/or development is inexplicit. Moreover, the current management of IVDD symptomatic patients only includes conservative measures and surgical intervention, with no cure or specific treatments being available [2].

Obesity, being itself one of the major public health problems in western society contributing to disability, has been implicated in the development of disc disease [4]. Besides the abnormal and excessive mechanical loading associated with being overweight, a biochemical link between obesity and IVDD has been proposed. The obese adipose tissue is characterized by an inflammatory environment and a deregulated production of cytokine-like hormones, adipokines [5], which have pleiotropic functions [6]. Adipokines are now recognized as important players not only in energy metabolism but also in immunity and inflammation, most of them contributing to the obesity-associated chronic low-grade inflammation [7]. Furthermore, adipokines have been implicated in the pathophysiology of rheumatic diseases, such as osteoarthritis (OA) and rheumatoid arthritis (RA), by influencing the pro-inflammatory environment within the joint, the cartilage catabolic activity, and both cartilage and bone remodeling [8,9].

In the intervertebral disc, inflammatory processes contribute to disc degeneration. In particular, exacerbated production of inflammatory mediators, to be pointed interleukin (IL)-1β, tumor necrosis factor (TNF)-α and IL-6, promoted matrix degradation, disc cell senescence, and death, as well as the recruitment of immune cells; altogether leading to the compromised biomechanical function of the intervertebral disc [10,11]. Despite the important role of adipokines in the immune system and in the pathophysiology of rheumatic diseases, whether adipokines act as mediators in IVDD remains largely unknown. Since leptin and its receptor have been identified in the human intervertebral disc [12,13], researchers aim to elucidate the role of adipokines in the development of IVDD.

This review provides a systematic overview of the current understanding of obesity, inflammation and IVDD interrelationships. In particular, adipokines are highlighted as important players in the disc degeneration pathophysiology, with emphasis on molecular mechanisms. Understanding the mechanisms of adipokines contribution to inflammatory, catabolic and nociceptive processes may hold therapeutic promise against IVDD.

## 2. Intervertebral Disc Degeneration (IVDD)

The intervertebral disc (IVD) is an important structural component that enables the bending, flexion, and torsion of the spinal column [14]. It comprises an inner cell-sparse gelatinous nucleus pulposus (NP), and the outer fibrous region of annulus fibrosus (AF), limited above and below by hyaline cartilaginous end plates (CEPs) [2,14]. The NP is a gel-like structure with chondrocyte-like cells characterized by an extracellular matrix (ECM) rich in proteoglycans, mainly type II collagen and aggrecan, which allows water retention. The hydrated, aggrecan-rich ECM of the NP creates a hydrostatic pressure to resist axial compressive loads from the trunk [2,11,14]. The AF is a lamellar fibrocartilagenous concentric ring with ECM-rich type I collagen, and with low proteoglycan content as well as water retention capacity. It is responsible for withstanding pressurized NP, and tensile and torsional pressures from adjacent vertebrae motion [2,11,14]. The CEP is a uniform thickness, homogenous hyaline cartilage that interfaces avascular disc tissue and blood supply, thus providing nutrients to disc cells. The ECM of CEP cells is formed mainly by proteoglycan and collagen fibers. In a normal adult intervertebral disc, homeostasis between ECM synthesis and degradation are kept in balance by a number of growth factors and cytokines. An imbalance towards catabolic processes contributed to the IVD structure degradation, with resulting disc degeneration and likely low back pain [2,11,14]. Understanding homeostatic and degenerative mechanisms of IVD are essential to disclose tissue biology and outline new therapeutic strategies.

**Figure 1:**
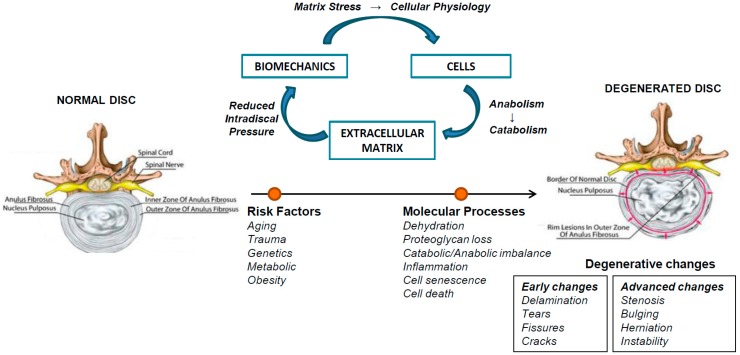
Pathophysiology of intervertebral disc disease. Homeostasis of the intervertebral disc depends on the interaction of biomechanical stress, cells and extracellular matrix. Several risk factors dysregulate this balance by triggering increased matrix degradation, angiogenesis/neovascularization, and enhanced expression of catabolic cytokines and nerve ingrowth. Decreased production of proteoglycans lead to a reduction in hydrostatic pressure and increase in shear forces leading to progressive degeneration.

IVDD is a chronic and irreversible process characterized generally by increased matrix degradation, loss of proteoglycans and hydration in NP, angiogenesis/neovascularization, nerve ingrowth, and expression of catabolic cytokines. These pathological features of IVDD lead to multiple anatomic, mechanical, and biochemical disc changes, including disc desiccation, disc bulging and reduced disc height, with resultant decrease of mechanical stability and shock absorber functions, which contributes to osteophyte formation, annular fissures, and decreased motion of spinal segments [2,15] (Figure 1). Since the affected spines are unable to withstand the normal loads, the adjacent tissues, especially ligaments and muscles, are also affected [2].

The etiology of IVDD has been related to aging, genetics, environmental and nutritional factors, as well as inflammation and increased cell senescence and apoptosis [2,3,15] (Figure 1). The young, healthy IVD is largely aneural and avascular excluding the outer third of AF. With aging, there is a progressive decrease of disc matrix proteoglycan content, a recognized inhibitor of vascular ingrowth, accompanied by degenerative anatomic modifications, like the growth of neuronal and vascularized granulation tissue into the inner layers of AF and NP. There is also a reduction in vascular ducts in CEP, which limits the diffusion of waste products out and nutrients into IVD. Moreover, aging augmented oxidative stress, mitochondrial dysfunction, DNA damage, cellular senescence and apoptosis, the levels of pro-inflammatory cytokines and damaged proteins, overall leading to disc matrix homeostasis imbalance and disc degeneration [3]. The changes in the structure of AF and NP significantly affects IVD mechanobiology, resulting in advanced disc degeneration [1,3]. Environmental risk factors like smoking, lack of exercise, unhealthy lifestyle, and occupational exposures (vibration, mechanical loading, and heavy trauma) have been described to contribute to IVDD pathogenesis [2,3]. Assessing the levels of trace elements in IVD, a higher copper concentration in disc tissue, compared to bone, was found, indicating the potential impact of environmental pollution in IVD. Since IVD is an avascular tissue, the higher copper concentration should be related to collagen and elastin fibers cross-linking rather than ceruloplasmin, albumins or oxygen transport [16]. Another important contributor to IVDD development is genetics, which is hypothesized to account to up 75% of IVDD etiology [17]. In fact, the IVDD onset has been associated with several polymorphisms in structural, degradative and inflammatory genes, including aggrecan (ACAN), collagen (COL I, IX and XI), hyaluronan and proteoglycan link protein 1 (HAPLN1), fibronectin (FN), asporin (ASPN), cartilage intermediate layer protein (CILP), thrombospondin (TBSP), vitamin D receptor (VDR), a disintegrin and metalloproteinase with thrombospondin motifs (ADAMTS)-4 and -5, matrix metalloproteases (MMP-1, -2, -3, -9 and -14), tissue inhibitor metalloproteinases (TIMPs), and transforming growth factor beta (TGF-β) gene polymorphisms [2,17]. Overall, IVDD is a complex multi-factorial pathology, and age-related, environmental and genetic risk factors are of potential interest in the prevention or treatment of this high incident disease.

Currently, IVDD is an untreatable, specific treatment-orphan disease. The available conservative treatments include bed rest, physiotherapy, and administration of analgesic and anti-inflammatory medications, such as non-steroidal anti-inflammatory drugs (NSAIDs), steroids, muscle relaxants, and opioids. Although these drugs allow for effective short-term back pain relief, the IVDD progression is not modified [2]. Additionally, some oral supplements, that is glucosamine or omega-3 fatty acids, have gained popularity in the last years, but some harmful effects have been suggested [3]. When conservative measures are inefficient and nerve compression, confirmed by radiographic imaging, is responsible for continuous pain sensation, the surgery is preferred. Interventional procedures, including fusion, discectomy, and total disc replacement, are highly intrusive and have a great risk of relapse, neighbouring segment degeneration and a loss of mechanical properties [2]. Both conservative treatments and surgical interventions are only directed to the clinical symptoms of the disease, being therefore largely ineffective and with no long-term action. In this context, novel therapeutic strategies targeting the IVDD pathophysiology at the molecular level have emerged. Protein-based, cell and gene therapies have been emerged as biological approaches to target, slow down or even revert disc degeneration [2,3]. Although cell and gene therapies are promising, further research and well-designed clinical trials are of utmost importance for its safe application on IVDD treatment [2,3].

## 3. Inflammation in IVDD

Degeneration of the intervertebral disc has been described to be associated with augmented levels of pro-inflammatory cytokines secreted by the disc cells or by infiltrating macrophages, neutrophils, and T cells [10]. During degenerative cascades, vascularization of inner AF and NP allows the migration of mast cells and macrophages into the disc, and the amplification or perpetuation of inflammatory cascades, with consequent induction of low back pain [3]. Secreted inflammatory mediators, in particular, TNF-α, IL-1α/β, IL-6, IL-8, IL-2, IL-17, IL-10, IL-4, IFN-γ, and PGE_2_ [10], promoted the ECM degradation, disc cell autophagy, senescence and apoptosis [2,10]. Cytokines levels were also correlated with pain sensation in patients, via upregulation of nitric oxide synthase and nitric oxide, which suggests a positive feedback loop of pain generation [3]. Moreover, pro-inflammatory cytokines induced the expression of neurogenic factors, namely nerve growth factor (NGF) and brain-derived neurotrophic factor (BDNF), which promoted the sprouting of nerve fibers from dorsal root ganglion (DRG) into AF and NP, and augmented nerve survival, as well as the action and sensitivity of nociceptive associated cation channels in DRGs [3,10]. Overall, chronic inflammation triggers irreversible structural and biochemical changes, including ECM degradation, as well as vascular and nerve innervation, that leads to IVDD pathophysiology and back pain [15].

The particular contribution of cytokines to pathological features of IVDD at cellular and tissue level were extensively reviewed previously [10,11], and thus, the contribution of TNF-α and IL-1β, the most studied cytokines, to IVDD pathophysiology, was briefly mentioned here. TNF-α was up-regulated in degenerated disc tissue, being associated with nerve ingrowth and irrigation, as well as disc herniation [10]. IL-1β, IL-1α, and the active receptor IL-1RI were also increased in IVDD, while the levels of its antagonist IL-1Ra were similar between normal and degenerated discs [18]. Revealing the importance of IL-1β in IVDD pathogenesis, the IL-1Ra deficient mouse model develops spontaneous disc degeneration [19]. Moreover, IL-1β polymorphisms were associated with increased risks of low back pain [14]. It was verified that both TNF-α and IL-1β, contributed to ECM degradation by the upregulation of catabolic mediators, including MMP-1, -2, -3, -13, -14, ADAMTS-4 and -5, and the suppression of matrix synthesis genes [10,11,18,20,21]. IL-1β also inhibited the synthesis of sulfate glycosaminoglycan, collagen type II and aggrecan, which can be reversed by hemeoxygenase-1 [22]. Additionally, TNF-α and IL-1β significantly augmented the expression of pro-inflammatory mediators, like IL-6, IL-8, IL-17, CCL3, iNOS, and prostaglandin-endoperoxide synthase 2 (PTGS2) in NP [23,24]. At a molecular level, most of the effects of TNF-α and IL-1β on the ECM were mediated via the heparan-sulfate proteoglycan syndecan-4 (SDC4), an activator of growth factors and MMPs, and the pro-inflammatory transcription factor nuclear factor (NF)-κB [10,11,21]. In particular, the p65 coactivator PHD3 (prolyl hydroxylase 3) was described to be involved in TNF-α-induced ECM catabolism [11].

## 4. IVDD and Obesity

Obesity, a public health epidemic in western countries, has largely been correlated with high-incident chronic inflammatory and autoimmune diseases, like type 2 diabetes mellitus, cardiovascular disease, Alzheimer’s disease or musculoskeletal pathologies [25]. Obesity has also been pointed as a mechanical risk factor for disc degeneration and low back pain in epidemiological studies [4,26,27]. Increased body weight and body mass index modify IVD biomechanics that, together with catabolic cell response and ECM degradation, represents a key factor in the induction of IVDD pathophysiology [1]. In fact, body weight has been associated with several features of IVDD, including disc space narrowing and reduced signal intensity of the IVD [28]. However, there are some contradictory data reporting that cumulative or repetitive loading associated with higher body mass have no deleterious action on IVD [29]. This suggests that beyond mechanical effects, obesity could exert metabolic and/or inflammatory effects that contribute to IVDD development [30,31].

**Figure 2:**
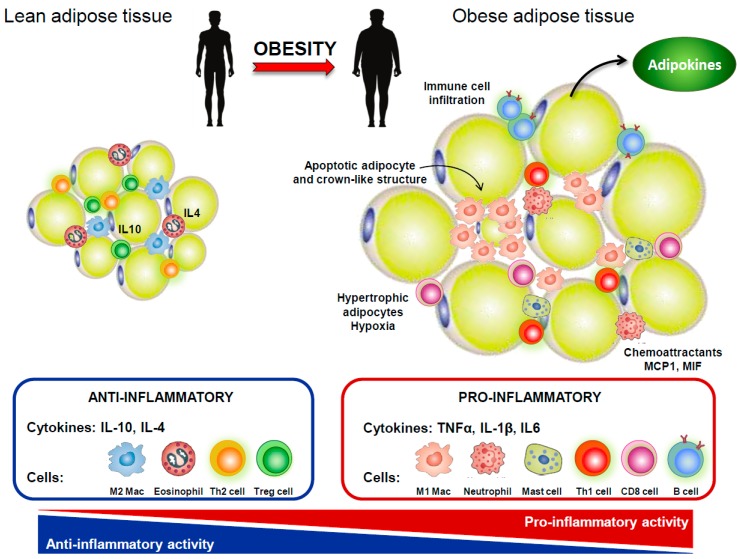
White adipose tissue as a pro-inflammatory tissue. In lean adipose tissue, the cross-talk between adipocytes and immune resident cells maintains tissue homeostasis. In particular, Treg cells secreted anti-inflammatory cytokines (IL10 and IL4) that promotes M2 macrophage phenotype. Overnutrition results in WAT expansion and adipocyte hypoxia, with consequent production of chemoattractants and infiltration of immune cells. B and T cells cells become activated, and there is a phenotypic switch from M2 to M1 macrophages, which accumulate around necrotic adipocytes forming ‘crown-like structures’. The deregulated production of adipokines and pro-inflammatory cytokines (TNF-α, IL-1β and IL-6) contributed to chronic low-grade inflammation.

**Figure 3:**
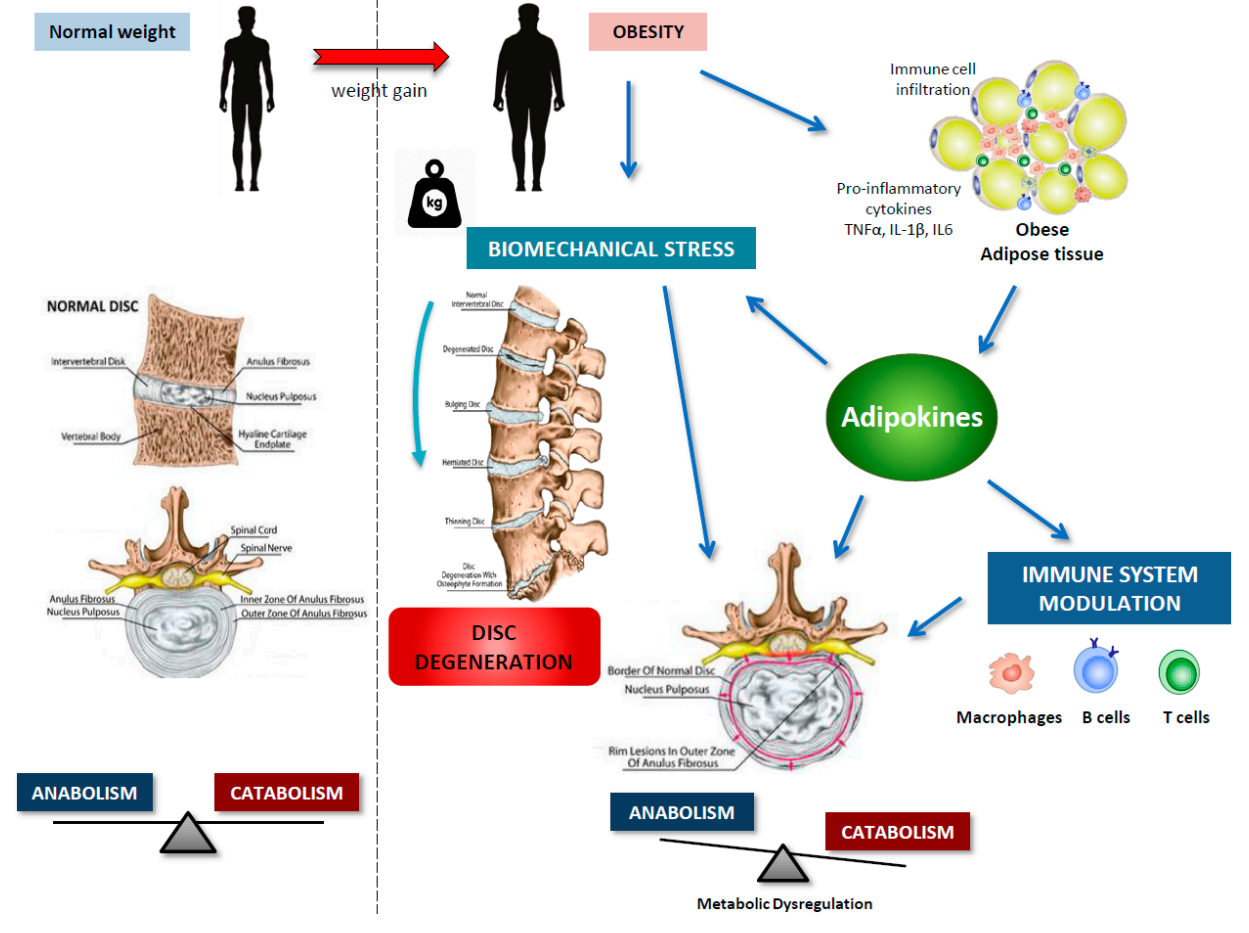
Adipokines as molecular linkers between obesity, immune system and intervertebral disc disease. Body weight gain, accompanied by white adipose tissue expansion, lead to chronic low-grade inflammation and increased biomechanical stress on intervertebral disc. Adipose tissue-derived adipokines cause imbalance of disc homeostasis towards catabolic processes and induce pro-inflammatory cytokine release from innate and adaptive immune cells, thus triggering degenerative pathways in intervertebral disc.

Adipose tissue is no longer viewed as a mere energy storage tissue; it is now realized as a dynamic endocrine organ composed not only by adipocytes but also by fibroblasts, endothelial cells, and several immune cells, particularly mast cells, neutrophils, eosinophils, adipose tissue macrophages, and B and T cells, which keep the homeostasis of adipose tissue in non-obese subjects [5,32]. Adipocyte expansion, resulting from a positive energy balance, is accompanied by adipocyte hypoxia, cell stress, and apoptosis, as well as increased expression of chemoattractant modulators that promote the infiltration of inflammatory cells to adipose tissue [5]. Furthermore, a deregulated production of cytokine-like hormones, i.e., adipokines, is observed in obese adipose tissue (Figure 2). These low molecular weight, biologically active peptides have demonstrated pleiotropic functions. By inducing anorexigenic factors and suppressing orexigenic signals at the hypothalamus, adipokines demonstrated an important role in energy metabolism by communicating the nutrient status of the organism [6]. Adipokines have also been highlighted as crucial regulators immune system response and inflammation [7]. Adipokines thus contributed to the obesity-associated chronic low-grade inflammation, and, as described above, inflammation played an important role in IVDD pathophysiology. Furthermore, adipokines have been revealed as crucial modulators of cartilage and bone homeostasis, contributing to the pathogenesis of musculoskeletal diseases, such as osteoarthritis and rheumatoid arthritis [8,9]. In the last years, adipokines have also been implicated in the pathophysiology of disc degeneration (Figure 3), with studies focusing on receptor identification, pathway analysis, and cellular proteome. Importantly, very recently, the hypertrophic vertebral marrow adipose tissue was pointed as a source of inflammatory adipokines that trigger degenerative pathways in IVD, via metabolism disturbance and the establishment of an initial inflammatory environment [33].

## 5. Adipokines in IVDD

### 5.1. Leptin

Leptin is a 16 kDa non-glycosylated cytokine-like hormone encoded by obese (ob) gene. It is mainly produced by white adipose tissue, but also by brain, placenta, skeletal muscle, intestines, bone and joint tissues [34]. The diabetes (db) gene encoded the leptin receptor (LEPR or OB-R), which exists in at least six isoforms (one soluble, four short and one long isoforms), differing in cytoplasmatic domain length. LEPR long isoform contains an intracellular domain that canonically transduces the leptin signal through the JAK/STAT pathway or alternatively via ERK1/2, JNK, p38 MAPK, PKC, or PI3K/Akt [35]. Considering the wide pattern of LEPR expression in peripheral tissues, leptin has demonstrated pleiotropic functions in physiological and pathological conditions. In particular, leptin has a crucial role in appetite and body weight homeostasis, through central signaling at the hypothalamus level, but has also been involved in the secretion of insulin, lipid homeostasis, thermogenesis, reproductive functions, angiogenesis, infection, inflammation, as well as bone and cartilage homeostasis [34]. In the recent years, leptin has been pointed out as an important linker between the neuroendocrine system, inflammation, and rheumatic diseases (OA and RA) [34]. In fact, obese, OA and RA patients demonstrated increased leptin levels. Moreover, several studies verified the expression of LEPR in osteoblasts and chondrocytes and reported leptin as a modulator of cartilage catabolic activity, joint pro-inflammatory environment, endochondral bone formation, and chondrocyte differentiation and mineralization [34]. Given the extensive data demonstrating the role of leptin in musculoskeletal diseases, namely OA and RA, potential involvement of this hormone in IVDD pathophysiology was hypothesized.

The expression of both leptin and its receptors (Ob-Ra and OB-Rb) were detected in intervertebral disc tissues, being augmented in the AF of grade II-IV degenerated discs [12,13]. It was also verified that a higher local leptin expression was found in the posterior compared to the anterior AF [36], and that 3D cultured AF cells demonstrated an abundant leptin production, which reveals a local autocrine or paracrine leptin regulatory system within disc cells [12]. Importantly, leptin and LEPR expressions were enhanced in cell clusters and fibrocartilaginous areas of IVD, which results from increased disc cell proliferation [12,13,37]. In fact, leptin directly stimulates the proliferation of NP and AF cells, by induction of cyclin D1 expression, which controls cell cycle transition from G1 to the S phase, and by activation of JAK/STAT3, MEK/ERK and PI3K/Akt signaling pathways in NP cells [37]. Of note, proliferating cells are unable to correctly synthesize ECM components, demonstrated increased expression of ECM degradative enzymes and thus, have enhanced catabolic metabolism, and partially contribute to disc cell senescence [13,37]. Therefore, altogether these findings indicated that the leptin-induced cell proliferation might be a fundamental mechanism, underlying disc degeneration. Moreover, leptin was evidenced to induce cytoskeleton remodeling by increasing β-actin expression and F-actin stress fiber formation in NP cells, via the Rho/ROCK/LIMK/cofilin pathway [38,39]. The altered expression and organization of cytoskeleton proteins, induced by leptin, may play an important role in the transduction of mechanical signals between IVD cells and surrounding ECM, hence contributing to the IVDD pathophysiology [38,39].

Analyzing the effects of leptin in disc cells proteome, recently, Segar and colleagues demonstrated that this adipokine, alone or in synergy with TNF-α, IL-1β, or IL-6, significantly increases nitric oxide production, and the expression of pro-inflammatory cytokines (TNF-α and IL-6), and MMPs (MMP-3, -7, -9, and -11, as well as ADAMTS -4, and -5) [31]. These data indicated that leptin can initiate degradative and inflammatory cascades in disc cells, being its deleterious effects potentiated by an existing inflammatory environment [31]. Similar effects were previously verified by Miao et al. in rat NP cells [40]. In particular, leptin, alone or in synergy with IL-1β, enhanced the expression of ADAMTS-4, and -5, COL2A1, MMP-1, and -13. Furthermore, leptin-mediated MMP-1 expression occurs via JAK2/SATAT3, ERK and JNK pathways, while MMP-13 expression is induced via JAK2/SATAT3, ERK, and p38 MAPK signaling pathways [40]. Li and co-workers also verified that leptin down-regulates both mRNA and protein levels of aggrecan, via p38 MAPK/ADAMTS pathway in human NP cells, thereby contributing to IVDD [41].

Recently, Han et al. analyzed the effects of leptin in the cartilaginous end plate (CEP) using a rat model of lumbar disc degeneration [42]. It was verified that leptin is greatly co-expressed with CEP calcification. In fact, leptin time- and dose-dependently augments osteogenic factors OCN and Runx2 expression, both markers of calcification, in rat CEP cells. Furthermore, leptin activates STAT3 and ERK1/2 signaling pathways that were demonstrated to be involved in leptin-induced OCN and Runx2 expression, and in the formation of mineralized nodules in CEP cells. Consequently, leptin can promote calcification of hyaline cartilage in CEP, interfering with nutrient transpor to the disc cells and, thus, lead to disc degeneration [42]. Leptin can also contribute to AF terminal differentiation, assessed by evaluation of specific differentiation markers (collagen X and MMP-13) expression, via activation of ERK1/2 and p38 MAPK, but not JNK1/2 [43]. Moreover, leptin can affect IVD adjacent tissues. In particular, leptin was described to be increased in lumbar spinal canal stenosis, to be positively correlated with ligamentum flavum hypertrophy and fibrosis, and to induce collagen I and III expressions, as well as IL-6 expression via NF-κB activation, in ligamentum flavum cells [44].

Altogether, the present data reveal the important role of leptin in the development of IVDD pathology by enhancing disc cells proliferation, remodeling of cell cytoskeleton, ECM degradation though augment of catabolic metabolism, pro-inflammatory cytokine production, and CEP calcification, among other poorly described or unknown mechanisms. However, further research in human IVD cells is needed.

### 5.2. Adiponectin

Adiponectin, also named AdipoQ, Acrp30, apM1, or GBP28, is a 244-aa adipokine encoded by ADIPOQ gene and structurally homologue to complement factor C1q, and collagen VIII and X. It is mainly produced by adipose tissue, but it is also secreted at lower levels by skeletal muscle, bone marrow, and cardiac tissue, being found in several molecular configurations (trimers, hexamers, and 12-18-monomers forms) [45,46]. Adiponectin exerts its biological functions by binding to two specific receptors: AdipoR1, mainly present in the skeletal muscle, and AdipoR2, prevalently found in the liver [47]. Transduction of adiponectin signal via these receptors involved the AMP-activated protein kinases (AMPK), peroxisome proliferator-activated receptor (PPAR)-α, or PPAR-γ pathways activation [46]. The adiponectin circulating levels tend to be down in morbidity obese individuals and in patients with metabolic syndrome or obesity-associated diseases, such as cardiovascular complications [46]. However, its plasma and serum concentrations were significantly elevated in cartilage and bone diseases, like osteoarthritis and rheumatoid arthritis [9]. In fact, adiponectin has been implicated in ECM degradation and thus cartilage destruction, via modulation of the immune system and MMPs expression and activity [9]. In the IVDD, the action of adiponectin remains largely unknown.

The adiponectin levels have been associated with disc degeneration. However, there are contradictory results. Khabour et al. found higher circulating adiponectin levels in patients suffering lumbar disc degeneration, compared with healthy controls [48]. By contrast, more recently, Yuan and colleagues verified a downregulation of adiponectin expression in NP cells from degenerated human IVD compared to healthy NP tissues [49]. Moreover, a negative correlation between adiponectin levels and IVDD severity was verified [49]. These apparent discrepancies could be attributable to differences in tissue samples and adiponectin sources. In fact, IVD is mainly an avascular tissue; thus, serum adiponectin concentration may be poorly related to IVD adiponectin levels [49]. It was hypothesized that decreased adiponectin amounts in IVDD are related to a reduction of viable NP cells and to impaired protein synthesis in senescent NP cells [49]. Since adiponectin has been realized as an crucialregulator of the immune response [9], its downregulation in IVD could lead to an imbalanced inflammatory response and thus, disc degeneration [49]. Furthermore, both healthy and degenerative NP cells secreted adiponectin, which indicates a local paracrine regulatory system [49]. The expression of adiponectin receptors was also investigated, but there are also conflicting results. Terashima and colleagues verified that AdipoR1 and AdipoR2 were gradually reduced with disc degeneration severity [50], whereas Yuan et al. reported an upregulation of both adiponectin receptors in degenerated IVD tissues and degenerated NP cells [49]. Likely, augmented AdipoR1 and AdipoR2 represent a compensatory mechanism to the decreased adiponectin expression aimed to increase tissue sensitivity to this adipokine and protect the disc from degeneration [49]. Concerning the effects of adiponectin in cell proteome, it was verified a downregulation of TNF-α secretion in human NP cells from degenerated discs in a dose- and time-dependent manner [49]. This was also verified in IL-1β-stimulated NP and AF cells from rat IVD tissue [50]. Adiponectin treatment had no effect on IL-6 expression in IL-1β stimulated IVD cells [50]. These findings indicated that adiponectin may act as an anti-inflammatory adipokine by suppressing the expression of pro-inflammatory mediators, including TNF-α, thus contributing to IVD homeostasis and protecting it from degeneration [49,50].

### 5.3. Resistin

Resistin, also called found in inflammatory zone 3 (FIZZ3) or adipocyte-secreted factor (ADSF), is a cysteine-rich 12.5 kDa protein found in human blood as dimers [51]. Although a specific resistin receptor was not been described yet, tool-like receptor 4 (TLR4) was demonstrated to mediate the resistin-induced secretion of pro-inflammatory factors [52]. Accordingly, resistin was initially described as a linker of obesity and diabetes by promoting insulin resistance, but in the last few years, it was also pointed out as a pro-inflammatory factor [8]. Lately, it was verified that resistin is up-regulated in serum and synovial fluid of OA and RA patients [53,54]. Moreover, resistin augmented many cytokines and chemokines expressions, in particular IL-12, IL-6, and TNF-α, via CCAAT/enhancer-binding protein (C/EBP)β and NF-κB [55], and its intra-articular injection induced the development of arthritis in healthy mouse joints [56]. It was also verified that resistin stimulates the migration of endothelial progenitor cells (EPCs) into synovium via vascular endothelial growth factor (VEGF), during RA angiogenesis [57]. Thus, resistin seems to be involved in musculoskeletal diseases pathology by modulating angiogenesis and inflammatory environment within the joint.

Healthy discs expressed low levels of resistin, that are increased during IVD degeneration, being positively correlated with Pfirrmann grade [58]. This data indicated that resistin could be involved in the development of disc degeneration. Liu and co-workers demonstrated that resistin time- and dose-dependently increased the expression of ADAMTS-5 in rat NP cells, being p38 MAPK signaling pathway involved [59]. Recently, Li et al. elucidated the role of resistin in IVDD [58]. It was verified that resistin augmented the expression of chemokine ligand 4 (CCL4, also named macrophage inflammatory protein, MIP-1β) in degenerated human nucleus pulposus tissues by direct binding to TLR4 [58]. Accordingly, it was previously demonstrated that TLR4 is expressed in IVD cells, being its activation associated with increased expression of inflammatory cytokines [60]. The resistin-induced CCL4 expression is mediated by NF-kB and p38-MAPK pathways but not by c-Jun N-terminal kinase (JNK) or extracellular signal-regulated kinase (ERK) pathways [58]. In particular, resistin-induced p38 and p65 phosphorylation and augment p65 binding to the promoter region of CCL4 gene [58]. Furthermore, resistin-treated rat NP cells promoted the macrophage migration, which could be blocked to some extent by CCL4-specific siRNA or by antagonizing the C-C chemokine receptor 1 (CCR1, the receptor of CCL4). Thus, in IVD tissue, resistin could bind TLR4 and increase CCL4 expression, via NF-κB and p38 MAPK activation, which promote the infiltration of macrophages [58]. However, further research is needed to fully understand the implications of this adipokine in IVD pathophysiology.

### 5.4. Progranulin

Progranulin (PGRN), also recognized as GP88, proepithelin, PC-cell-derived growth factor, granulin-epithelin precursor (GEP), or acrogranin, is a 68-88 kDa cysteine-rich, secreted glycoprotein that can be subjected to enzymatic proteolysis, originating small homologous subunits, in particular granulins and epithelins [61]. Lately recognized as an adipokine, PGRN is produced by several cells, including macrophages, epithelial cells, and chondrocytes [9]. It was verified that PGRN directly interacts with TNF receptors [61,62]; PGRN and TNF-α showed similar binding affinity to TNFR1 (associated with pro-inflammatory activity), whereas PGRN showed much greater affinity than TNF-α to TNFR2 (linked to immunosuppressive effects). Therefore, PGRN has been implicated in several physiological and pathological processes like inflammation, wound healing, obesity and rheumatic diseases [9]. Accordingly, PGRN was reported to be expressed in human articular cartilage, being its circulating and synovium levels augmented in OA and RA patients [9]. Its mechanisms of action include modulation of chondrocyte proliferation and differentiation, anti-inflammatory and anti-degradative activity by inducing anabolism via TNFR2 activation and by diminishing catabolism through TNFR1 binding and blocking of TNF-α-induced effects, and regulation of endochondral ossification of growth plate during development [9].

PGRN expression is increased in peripheral blood sera and disc tissues of IVDD patients, and its blood levels were positively correlated with clinical symptoms [63]. Progranulin protein levels also increase after spinal cord contusion, being colocalized with activated microglia and macrophages [64]. Demonstrating the potential action of PGRN in degenerative processes of IVD, it was verified that PGRN loss accelerated IVD degeneration in aging mice [65]. Most of the evidence on the contribution and action mechanism of PGRN on IVDD pathophysiology were obtained using PGRN knockout mice [65]. In particular, PGRN-/-mice significantly exhibits: (i) higher levels of osteoblast gene markers and osteoclast activity; (ii) proteoglycan loss, especially in AF and CEP; (iii) degradation of aggrecan; (iv) increased levels of ADAMTS-5 and MMP-13; (v) dramatic augment of pIκB-α levels; (vi) elevated levels of iNOS, both mRNA and protein; and (vii) greater activation of β-catenin signaling pathway, evidenced by higher levels of mRNA, protein, nuclear translocation and downstream proteins, namely Axin2 and RUNX2 [65]. Furthermore, evaluating the effect of aging in disc degeneration pathophysiology, Zhao and co-workers evidenced that degeneration process occurred earlier in CEP tissue, which may accelerate AF and NP degeneration since newly formed bone tissue in degenerated CEP would seriously affect the diffusion of nutrients to AF and NP [65]. More recently, Wang et al. associated PGRN with IVDD through modulation of IL-10 and IL-17 cytokines [63]. In particular, the authors hypothesize that PGRN effect on IVDD may depend on: (i) PGRN interaction with TNFR1 and thus, protection from TNF-α-mediated degradation; (ii) decrease of IL-17 expression via TNFR1, abolishing Th17 recruitment and immune responses; and (iii) PGRN binding to TNFR2 with consequent IL-10 production and anti-inflammatory activity [63]. Furthermore, using the PGRN derived engineered protein atsttrin, Ding and colleagues, verified an inhibition of TNF-α-mediated degenerative processes in IVD [66]. In particular, atsttrin strongly inhibited TNF-α-induced MMP-13, iNOS, COX-2, IL-6, and IL-17 expression in human NP cells [66]. Collectively, these data revealed PGRN as a crucial player in maintaining IVD homeostasis, likely through suppression of NF-κB and β-catenin signaling pathways, which prevent abnormal bone metabolism and degeneration of cartilage-like tissue in IVD [65]. Furthermore, the PGRN engineered protein atsttrin was evidenced as a novel drug candidate for disc degeneration via targeting of TNF-α. However, further studies in human IVD tissue are needed.

### 5.5. Visfatin

Nicotinamide phosphoribosyltransferase (NAMPT), also named as visfatin or pre-B cell colony-enhancing factor (PBEF), is a homodimeric 52 kDa cytokine-like peptide that acts as both extracellular (eNAMPT) and intracellular (iNAMPT) forms [67]. NAMPT is the rate-limiting enzyme in the biosynthesis of nicotinamide adenine dinucleotide (NAD) from nicotinamide and is likely to be involved in cell differentiation, stress response, and apoptosis. Furthermore, visfatin levels were enhanced in metabolic pathologies and inflammation, but its function is still ill-defined [8]. At cartilage, visfatin was described to increase the production of pro-inflammatory mediators, like PGE_2_, and to induce ECM degradation by modulation of MMPs, ADAMTS, collagen 2 and aggrecans expression [8]. Visfatin also osteogenic differentiation and osteoblast proliferation [8]. In fact, serum and synovium visfatin levels were augmented in OA and RA patients, which indicates that visfatin can play a crucial function in the pathophysiology of musculoskeletal diseases [8].

In intervertebral disc, Shi et al. recently reported increased levels of visfatin in NP tissue from severe IVDD grades (Pfirrman grade IV and V), compared with low grades (Pfirrman grade II and III) samples [68]. In NP cells, IL-1β time- and dose-dependently augmented the expression of visfatin, which is involved in the IL-1β-mediated decrease of ECM-related proteins expression, aggrecan and collagen 2, and augment of degradative-associated proteins, ADAMTS4/5 and MMP-3/13, demonstrated by the use of visfatin inhibitor APO866 and visfatin knockdown with shRNA [68]. Inhibition of visfatin also induced NP cell autophagy, assessed by determination of beclin-1 and LC3-I/LC3-II levels [68]. Of note, autophagy inhibitor 3-methyladenine blocked the action of visfatin inhibitor on ECM-related proteins [68]. Altogether, these data revealed visfatin as an important player in disc degeneration and pointed out that visfatin inhibition protects NP cells from degeneration, thus having therapeutic potential in the alleviation of IVDD.

### 5.6. Lipocalin-2

Lipocalin-2 (LCN2; also recognized as human neutrophil lipocalin, neutrophil gelatinase-associated lipocalin, siderocalin, uterocalin, α-1-microglobulin-related protein, migration-stimulating factor inhibitor, 24p3, or p25), is a multifunctional 25 kDa glycoprotein expressed in kidney, human neutrophil granules, immune cells, spleen, liver, chondrocytes, and most significantly white adipose tissue, its major source [9]. Mouse LCN2 was reported to bind transporter protein SLC22A17 (24p3R), while human LCN2 binds megalin/glycoprotein GP330, an LDL receptor [9]. LCN2 have demonstrated regulatory roles in the hematopoietic cells apoptosis, immune system response and metabolic homeostasis. In the last years, LCN2 has been described as a sensor of mechanical load and inflammatory status of the joint, contributing to deregulation of cartilage and subchondral bone homeostasis, as well as bone-cartilage crosstalk, thus being implicated in OA and RA pathophysiology [8,9]. However, the role of LCN2 to disc degeneration remains largely unknown. Kao and colleagues demonstrated that nerve growth factor (NGF), an IVDD inducer, up-regulates the expression of LCN2, which forms covalent complexes with MMP9, blocking its auto-degradation and hence, increasing its activity, in rat AF cells [69,70]. In fact, NGF increased the expression of MMP9 protein but not gene expression in AF cells [69]. These results indicated that LCN2 could be implicated in disc degeneration, but further studies are needed.

### 5.7. Ghrelin

Ghrelin is a 28-residue peptide hormone initially described to be secreted by stomach’s oxyntic glands but is also expressed in lung, hypothalamus, ovary, testis and pancreatic islets [71,72]. Acting via growth hormone secretagogue receptor (GHSR), this hormone exerts pleiotropic paracrine/autocrine functions, including growth hormone secretion, and modulation of reproductive axis, adiposity, food intake, glucose metabolism, and motility of the gastrointestinal tract [71,72]. Thus, ghrelin has been implicated in physiological as well as pathological processes, like gastric ulcer, tissue repair, tumorogenesis and chondrogenesis [71,72,73]. Furthermore, ghrelin was reported as an anti-inflammatory hormone by inducing M2 macrophage profiling and inhibit M1 macrophages, as well as by inhibiting Th1 cells and increasing polarization of Th2 and regulatory T cells [74].

Recently, Li et al. elucidated the role of ghrelin in NP degeneration [75]. Ghrelin was detected in human NP tissue as well as in human primary NP cells. Furthermore, its levels were decreased in isolated NP cells following IL-1β treatment; thus, suggesting a potential role of ghrelin in NP cells degeneration [75]. In particular, ghrelin treatment down-regulates NP catabolism and inflammation by reducing IL-1β-induced ADAMTS-5, MMP-13, iNOS and TNF-α in NP cells, as well as ADAMTS-4, ADAMTS-5 and MMP-13 in a rabbit IVD degeneration model [75]. Ghrelin also protects NP tissue from degeneration by inducing the production of ECM components, to be pointed aggrecan, collagen 2 and Sox-9, in a rabbit IVD degeneration model [75]. Moreover, ghrelin reversed the IL-1β-stimulated apoptosis and disorganized proliferation in NP cells [75]. Studying the ghrelin mechanisms of action, it was verified that this hormone induces the production of ECM components through interaction with its receptor GHSR [75]. Ghrelin is also able to inhibit NF-kB signaling pathway by decreasing IκB phosphorylation and p65 nuclear transactivation [75]. Furthermore, ghrelin-induced Akt activation is implicated in the anabolic properties of this hormone in NP cells [75]. Altogether, these data indicated the protective role of ghrelin in disc degeneration by maintaining the homeostasis between anabolic and catabolic/inflammatory processes in NP cells.

## 6. Conclusions

The available evidence summarized here, indicates that obesity, beyond mechanical action, exerts important metabolic and inflammatory effects on the homeostasis of intervertebral discs, which are mediated by adipokines (Figure 3). However, most of the studies are focusing on leptin with little research evaluating the role of adipokines with demonstrated actions in osteoarthritis and rheumatoid arthritis. Furthermore, most of the studies are conducted in murine cells or in tissues/cells from surgical patients with little or no information on clinical IVDD aspects (for example degenerative Pfirrmann grade). It is also important to recognize that IVDD is a complex and multi-factorial disease and thus, further basic and clinical research is needed to fully characterize the multifaceted actions and molecular signaling pathways of adipokines in this pathology. Understanding the underlying detrimental effects of obesity-related molecules, in particular, adipokines, on IVDD pathophysiology, will certainly provide new therapeutic routes for this high-prevalent and disabling musculoskeletal disease.

## Abbreviations

IVDD	Intervertebral disc (IVD) degeneration
OA	Osteoarthritis
RA	Rheumatoid arthritis
IL	Interleukin
TNF	Tumor necrosis factor
NP	Nucleus pulposus
AF	Annulus fibrosus
CEP	Cartilaginous end plate
ECM	Extracellular matrix
MMP	Matrix metalloprotease
ADAMTS	A disintegrin and metalloproteinase with thrombospondin motifs
NOS	Nitric oxide (NO) synthase
JAK	Janus kinase
STAT	Signal transducer and activator of transcription
ERK	Extracellular-signal-regulated kinase
JNK	c-Jun N-terminal kinase
MAPK	Mitogen Activated Protein Kinase
PI3K	Phosphoinositide 3-kinase
LEPR	Leptin receptor
PGRN	Progranulin
TNFR	TNF-α receptor
LCN2	Lipocalin-2
